# Functional Characterization of Outer Membrane Proteins (OMPs) in *Xenorhabdus nematophila* and *Photorhabdus luminescens* through Insect Immune Defense Reactions

**DOI:** 10.3390/insects10100352

**Published:** 2019-10-17

**Authors:** Reyhaneh Darsouei, Javad Karimi, Gary B. Dunphy

**Affiliations:** 1 Biocontrol and Insect Pathology Lab., Department of Plant Protection, School of Agriculture, Ferdowsi University of Mashhad, Mashhad 9177948974, Iran; rdarsouei@gmail.com; 2 Department of Natural Resource Sciences, Faculty of Agricultural and Environmental Sciences, McGill University, 21 111 Lakeshore Rd, Ste. Anne de Bellevue, QC H9X 3V9, Canada; gary.dunphy@mcgill.ca

**Keywords:** antimicrobial peptides, cellular defense, insect pathology, phenoloxidase, phospholipase A2, protease

## Abstract

*Xenorhabdus nematophila* and *Photorhabdus* *luminescens* are entomopathogenic bacterial symbionts that produce toxic proteins that can interfere with the immune system of insects. Herein, we show that outer membrane proteins (OMPs) could be involved as bacterial virulence factors. Purified totals OMPs of both bacterial species were injected into fifth instar larvae of *Spodoptera* *exigua* Hübner. Larvae were surveyed for cellular defenses fluctuations in total haemocyte counts (THC) and granulocyte percentage and for the humoral defenses protease, phospholipase A2 (PLA_2_), and phenoloxidase (PO) activities at specific time intervals. Changes in the expression of the three inducible antimicrobial peptides (AMPs), cecropin, attacin, and spodoptericin, were also measured. Larvae treated with OMPs of both bacterial species had more haemocytes than did the negative controls. OMPs of *X. nematophila* caused more haemocyte destruction than did the OMPs of *P. luminescens*. The OMPs of both bacterial species initially activated insect defensive enzymes post-injection, the degree of activation varying with enzyme type. The AMPs, attacin, cecropin, and spodoptericin were up-regulated by OMP injections compared with the normal larvae. The expression of these three AMPs was maximal at four hours post injection (hpi) with *P. luminescens* OMPs treatment. Expression of the three AMPs in *X. nematophila* treated insects was irregular and lower than in the *P. luminescens* OMPs treatment. These findings provide insights into the role of OMPs of entomopathogenic nematode bacterial symbionts in countering the physiological defenses of insects.

## 1. Introduction

*Xenorhabdus nematophila* and *Photorhabdus luminescens* are gram-negative bacteria (Family Enterobacteriaceae) symbiotically associated with the entomopathogenic nematodes (EPNs), *Steinernema carpocapsae* Weiser, and *Heterorhabditis bacteriophora* Poinar, respectively [1]. The EPN infective juvenile stage (IJ) harbors the bacteria in their intestine, releasing them into the haemocoel of the host, causing insect death within 24–48 h post infection [2]. Several bacterial insecticidal factors characterized in *X. nematophila* and *P. luminescens* (Txp40 toxin, Tc toxin, 17-kDa pilin protein) have important roles bacterial virulence and hence EPNs efficacy [3,4], including the pilin protein overcoming host immune activities [5].

The pathogenicity of some Gram-negative bacteria depends on their ability to secrete virulence factors into the mammalian host by releasing outer membrane vesicles (OMVs) [6]. Some OMVs virulence factors include phospholipase C, proteases, elastases, hemolysins [6], phospholipids, lipopolysaccharides (LPS) (also known as endotoxins [2]), alkaline protease, and outer membrane integrated membrane proteins (OMPs). In pathogenic bacteria, some OMPs have been identified as virulence factors helping the bacteria escape avoid host defense mechanisms [7]. Inducible OMPs in *Xenorhabdus* and *Photorhabdus* were identified, including the stress response proteins *skp* in *P. temperata* [8]. Opns, an inducible protein of *X. nematophila* produces a growth advantage in insect hemolymph [9]. Major defensive factors of insect immune systems are the interactive cellular (haemocyte) and humoral elements. In *S. exigua*, the major haemocyte types reacting against bacteria include the granulocytes and plasmatocytes [10] which respond to particulate antigens by phagocytosis and nodulation [11]. Humoral factors in this insect species include the synthesis of antimicrobial peptides (AMPs) e.g., cecropins, attacins, the pattern recognition protein lysozyme [12], activation of the prophenoloxidase cascade and phospholipase A_2_ (PLA_2_) [11].

Due to limited functional information about OMPs of *X. nematophila* and *P. luminescens*, the current study was designed to survey the effects of the OMPs on aspects of cellular and humoral defensive enzymes in the haemolymph of *S. exigua* larvae. We surveyed total haemocyte counts (THC), differential haemocyte counts (DHC), and protease, phospholipase A_2_ (PLA_2_), and phenoloxidase (PO) activity, as well as the expression patterns of cecropin, attacin, and spodoptericin in response to exposure to total purified OMPs of *X. nematophila* and *P. luminescens.*

## 2. Materials and Methods

### 2.1. Insect Culture

Different larval stages of *Spodoptera exigua* collected from the sugar beet fields at [Mashhad, Razavi Khorasan province (36° 29′ N, 59° 60′ E), Northeastern Iran] were reared (under a 16:8 (L:D) h photoperiod, at R.H. 60 ± 5% and 28 ± 1 °C in the laboratory) on sugar beet leaves. Moths were fed with 20% honey solution. The eggs were collected daily. One-day-old larvae were fed with fresh sugar beet leaves and the fifth instar larvae (0.78 ± 0.026 mg weight) were used for the experiments.

### 2.2. Purification of Outer Membrane Proteins (OMPs) 

#### 2.2.1. Bacteria Growth 

*Photorhabdus luminescens* and *X. nematophila* were isolated from the nematodes *H. bacteriophora* and S. *carpocapsae* nematodes (supplied by e~nema GmbH company, Schwentinental, Germany), respectively. Bacterial stocks were cultured on NBTA medium containing nutrient agar, triphenyl tetrazolium chloride (0.004% wt/vol), and bromothymol blue (0.0025% wt/vol). For experimental purposes, a 48 h old colony was transferred into the 100 mL nutrient broth (NB) medium in a 500 mL Erlenmeyer and incubated (at 28 ± 1 °C, 120 rpm) for 24 h. The bacteria were cultured again by adding the 100 mL culture to a l liter volume nutrient broth medium in 5 Erlenmeyer flask with 500 mL volume) and shaken at 100 rpm on a horizontal shaker (48 h at 28 ± 1 °C).

#### 2.2.2. Preparation of OMPs from Symbiotic Bacteria

The OMPs were prepared from the culture supernatant as described by Korhonen et al. [13] with modifications. Briefly, after growth for 48 h in NB, the cells were collected by centrifugation (15 min at 4000× *g*). The pellets were suspended in TEB buffer (1 mM benzamidine, 1 mM EDTA pH, 8, 5 mM Tris-HCl pH, 8) and homogenized by micropestle. Cell debris was collected by centrifugation (5 min at 2000× *g*) and the supernatant removed. The OMPs in the supernatant were precipitated by adding crystalline ammonium sulfate to 50% saturation followed with incubation at 4 °C overnight. The precipitate was collected by centrifugation (1 h at 10,000× *g*), dissolved in 1 mL of 5 mM Tris buffer, and then dialyzed for 48 h against 5 mM Tris buffer (100 mL). Sodium deoxycholate (DOC) (0.5% *w*/*v*,) was added to the suspension, which was then dialyzed against 5 mM Tris buffer (100 mL) containing sodium deoxycholate (0.5% *w*/*v*) for 48 h. The suspension was centrifuged for 10 min at 10,000× *g*. The pellet contained DOC-insoluble material (outer membrane proteins). OMPs (5 mg) were dissolved in 1 mL of TENS buffer (50 mM Tris-HCl [pH 7.2], 400 mM NaCl, 5 mM EDTA, 1% sodium dodecyl sulfate). The concentration of OMPs were determined by the Bradford method using the standard curve of BSA. Their molecular weight was estimated by sodium dodecyl sulfate polyacrylamide gel electrophoresis (SDS-PAGE). SDS-PAGE was stained with Coomassie Blue dye. 

### 2.3. Injection of Bacterial OMPs

The fifth instar larvae of *S. exigua* were injected with 5 µL OMPs (0.6 mg/mL) in TENS buffer using an insulin syringe (30 G, B. Braun; Melsungen, Germany). Negative control larvae received 5 µL TENS buffer. In the gene expression experiment, a treatment without injection was considered as the normal sample. Larvae were subsequently kept at room temperature and fed with fresh leaves. The different aspects of the immune defenses were surveyed using at 0.5, 2, 4, 8, 12, and 16-h post-injection (hpi). 

### 2.4. Total Haemocyte Counts and Differential Haemocyte Counts (THC and DHC)

For THC assay, the surface of injected larvae was disinfected with 70% ethanol. Five µL of hemolymph were collected by cutting the prothoracic leg. Haemocytes were counted on a Neubauer hemocytometer (Marienfeld, Lauda-Königshofen, Germany). Haemocyte numbers were calculated based on Jones’s formula [14]. For DHC, haemolymph (10 µL) was smeared on the glass microscope slide. The cells were fixed with acetic acid: methanol (1:3 *v*/*v*) for 5 min [15] and stained with 10% (*v*/*v*) Giemsa [16]. One hundred haemocytes were counted randomly and haemocyte types were determined according to Ribeiro and Brehelin [17] and recorded as a percentage of total cells. 

### 2.5. Protease Assay

For total protease activities of *S. exigua* larval haemolymph, azocasein (Sigma, Taufkirchen, Germany) was used as a substrate and the absorbance was determined at 450 nm on a microplate reader (Stat Fax 3200^®^; Awareness Technology Inc., Palm, FL, USA). 48.5 µL Tris buffer (pH 9), 16.5 µL azocasein 3%, and 10 µL haemolymph were kept at 37 °C for 2 h. After adding 50 µL of 30% trichloroacetic acid, the protease activity was stopped. The samples were incubated at 4 °C for 30 min, the centrifuged at 10,000× *g* for 10 min and 100 µL of the supernatant mixed with NaOH 1 M (100 µL). The activity of protease was expressed as µmol dye/min/mg protein using the extinction coefficient of the chromogenic azo group produced by the cleavage of casein [18].

### 2.6. Phospholipase A_2_ (PLA_2_) Assay

The PLA_2_ was assayed using a modification of Radvanyi et al. [19]. Pyrene-labeled phospholipid (Sigma) was used as a substrate. The PLA_2_ activity was calculated by spectrofluotometry (Cecil CE9500, Millersville, PA, USA) and the fluorescence intensity recorded using excitation and emission wavelengths of 345 and 398 nm, respectively. 

### 2.7. Phenoloxidase (PO) Assay 

For phenoloxidase activity, L-dihydroxyphenylalanine (L-Dopa, Sigma) was used as a substrate. Hemolymph was centrifuged (2000× *g*, 4 °C 1 min) and the supernatant plasma was used as the enzyme source. One hundred µL L-Dopa (60 mM), 90 µL phosphate buffer (pH 8.6) and 10 µL supernatant were added to microplate wells. An increase in absorbance was recorded every 30 s over 5 min at 492 nm using a Stat Fax 3200 Microplate Reader. One unit of PO activity was defined as the amount of enzyme that oxidizes 1 mol of L-Dopa per min per mg total plasma protein at room temperature (25 ± 2 °C). Total protein concentration was estimated according to the Bradford method [20]. Different concentrations of BSA were used in the standard curve [21]. 

### 2.8. Gene Expression

#### 2.8.1. RNA Extraction and cDNA Synthesis

Total RNA was extracted from haemolymph of the larvae at 2, 4, 8, and 16-h post injection using RNA extraction kit (catalogue number A101231 following the manufacturer’s instructions, Pars tous company, Tehran, Iran) then were treated with DNase I (catalogue number MO5401, Sina colon company, Tehran, Iran) according to the manufacturer’s instructions. The first-standard cDNA was synthesized with 1 µg of total RNA, oligo-dt primers, and reverse transcriptase according to the manufacturer’s protocol. 

#### 2.8.2. Design and Synthesis of Primers 

For AMPs expression of the target genes, attacin, cercopin, and spodoptericin, a set of primers were designed and used. Elongation factor 2 (EF2) gene was the reference gene used for normalization [22].

#### 2.8.3. Quantitative PCR (qPCR) 

qRT-PCR reactions were carried out in optical 8-cap strips (BioRad, California, CA, USA) on a BioRad machine model CFX96 using the 2X SYBR Green master mix. The total reaction volume (20 μL) contained 10 μL of SYBR Green, 0.2 μL of both the forward and reverse primers, and 2 μL of cDNA (1 µg concentration). The PCR conditions consisted of 95 °C for 10 min; 40 cycles of 95 °C for 15 s, 65 °C for 30 s, and 72 °C for 30 s, followed by a melt curve analysis at 95 °C for 10 s. Subsequently a temperature transition rate of 0.5 °C/s was performed from 50 to 95 °C. The cycle threshold (CT) values were determined by CFX96 software. The relative expression ratios of the target gene in treated groups were calculated using a 2^−ΔΔCT^ method [23]. All assays were performed on two independent replicates.

### 2.9. Statistical Analysis

The data were analyzed using two-way ANOVA (SAS Institute, [24]). Here, OMPs and time intervals were the two main effects. The effect of either factor alone and interactive effect (OMPs types × times interval) were calculated. When a significant result for the ANOVA was obtained (*p* < 0.05), a slicing test was used to measure the significant difference between means. Prior to ANOVA analysis, data were assessed for the assumption of ANOVA normality and homogeneity of variance (SAS Institute, [24]). All graphic data indicate the mean ± standard error (SE) of the mean in each treatment. The experiments were done at least two times with four insects for each experiment.

## 3. Results 

### 3.1. Outer Membrane Protein Profiles

**Figure 1:**
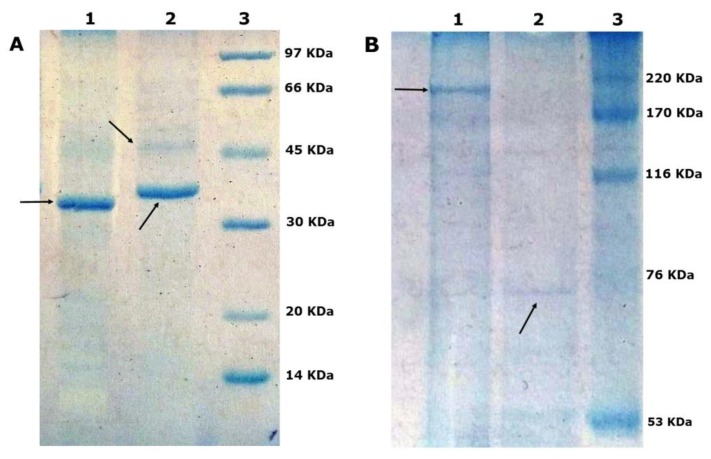
Coomassie blue dye stained SDS-PAGE gels showing the outer membrane proteins, (1) *Xenorhabdus nematophila*, (2) *Photorhabdus luminescens*, (3) Standard. (**A**) The molecular weight 14–97 kDa, (**B**) The molecular weight 53–220 kDa. Protein fragments have shown with arrows.

The OMPs profiles of *X. nematophila* and *P. luminescens* differed. The protein diversity in *X. nematophia* was more than that of *P. luminescens* (Figure 1). Protein with a molecular mass of ~35–40 kDa was more abundant of *X. nematophila* than other proteins. The total extracted protein concentration for the same amounts of *X. nematophila* and *P. luminescens* was 1.075 and 1.554 mg/mL, respectively. Also, the 260/280 ratio in *X. nematophila* and *P. luminescens* was 1.12 and 1.07, respectively. The concentration of OMPs in *X. nematophila* and *P. luminescens* using Bradford method was 0.84 ± 0.1 and 0.64 ± 0.1 (mg/mL), respectively.

### 3.2. Total Haemocyte Count

**Figure 2:**
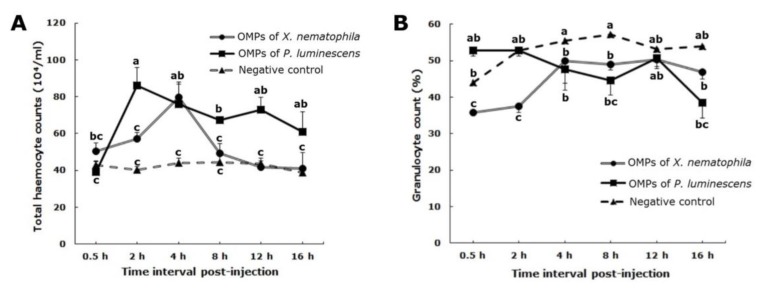
Changes in the patterns of cellular defense elements in fifth instar larvae of *Spodoptera exigua* after injection with outer membrane proteins of either *Xenorhabdus nematophila* or *Photorhabdus luminescens*, (**A**) total haemocyte count, (**B**) granulocyte percentage. Each measurement consists of eight replications. The vertical bars represent the standard error of the means. Different letters above the error bars indicate a significant difference of interactive effect means between outer membrane proteins from the two bacterial species × interval times at α = 0.05 (Slicing test).

There were differences in effects total of OMPs on THC depending on the bacterial species and incubation times (F_10,126_ = 3.37, *p* < 0.05). The OMPs (F_2,126_ = 24.61, *p* < 0.05) and time post-injection (F_5,126_ = 5.67, *p* < 0.05) significantly affected THC values (Figure 2A). In larvae with *P. luminescens* OMPs, the haemocytes levels were generally greater than those caused by *X. nematophila* OMPs, however, the rate of the decline in THC in larvae with *X. nematophila* OMPs was faster than those with *P. luminescen* OMPs (Figure 2A). Compared with the control treatment constant THC values, the THC levels in larvae with *P. luminescens* OMPs increased to maximum level at 2 hpi (2 h: F_1,14_ = 22.11, *p* < 0.05) then decreased from 4–16 hpi (4 h: F_1,14_ = 7.81, *p* < 0.05; 8 h: F_1,14_ = 34.71, *p* < 0.05; 12 h: F_1,14_ = 17.53, *p* < 0.05; 16 h: F_1,14_ = 3.89, *p* < 0.05). With the exception of 0.5 hpi, *P. luminescens* OMPs elevated THC above the control counts. *X. nematophila* OMPs elevated THC levels to a maximum count by 4 hpi (4 h: F_1,14_ = 16.91, *p* < 0.05) more slowly than did *P. luminescens* OMPs, the levels then decreased. There was a significant difference between *X. nematophila* OMPs and control treatment in THC density by 0.5 and 4 hpi (0.5 h: F_1,14_ = 2.2, *p* < 0.05; 2 h: F_1,14_ = 16.46, *p* < 0.05; 4 h: F_1,14_ = 16.91, *p* < 0.05). While in larvae with *P. luminescens* OMPs, a significant difference was observed compared with the negative control occurred from 2 to 16 hpi (2 h: F_1,14_ = 22.11, *p* < 0.05; 4 h: F_1,14_ = 7.81, *p* < 0.05; 8 h: F_1,14_ = 34.71, *p* < 0.05; 12 h: F_1,14_ = 17.53, *p* < 0.05; 16 h: F_1,14_ = 3.89, *p* < 0.05) (Figure 2A). 

### 3.3. Granulocyte Counts

There was a significant interactive effect on granulocytes between OMPs of both bacterial species over time (OMPs × times) (F_10,126_ = 2.76, *p* < 0.05). Although the granulocyte percentages between OMPs types were not significantly different (F_2,126_ = 1.26, *p* > 0.05), analysis of granulocytes data over time intervals (F_3,126_ = 148.46, *p* < 0.05) indicated there was a significant difference among specific times (Figure 2B).

In larvae with *P. luminescens* OMPs, the granulocyte percentages from 4 to 16 hpi were less than the negative control values (4 h: F_1,14_ = 4.87, *p* < 0.05; 8 h: F_1,14_ = 3.09, *p* < 0.05; 12 h: F_1,14_ = 1.37, *p* > 0.05; 16 h: F_1,14_ = 0.48, *p* > 0.05). The percentage of granulocytes in larvae with OMPs of *X. nematophila* was always less than the negative control. However, the granulocyte percentage for both spp. increased to a maximum plateau density by 4 hpi (0.5 h: F_1,14_ = 0.01, *p* > 0.05; 2 h F_1,14_ = 2.66, *p* > 0.05; 4 h: F_1,14_ = 1.57, *p* > 0.05). There was a significant difference in the number of granulocytes by 0.5 hpi after injection OMPs of *X. nematophila* and *P. luminescens* (0.5 h: F_1,14_ = 15.85, *p* < 0.05; 2 h: F_1,14_ = 3.77.1, *p* > 0.05; 4 h: F_1,14_ = 0.06, *p* > 0.05; 8 h: F_1,14_ = 0.95, *p* > 0.05; 12 h: F_1,14_ = 0.02, *p* > 0.05; 16 h: F_1,14_ = 3.27, *p* > 0.05) (Figure 2B).

The changes in levels of other haemocytes, including plasmatocytes, spherulocytes, and oenocytoides, were calculated. In larvae with *X. nematophila* OMPs, plasmatocytes, and spherulocytes percentages were more than those in the negative control insects, their trends in during time were irregular. There were no oenocytoids in the haemolymph. In larvae with *P. luminescens* OMPs treatments, the highly irregular fluctuation plasmatocyte densities at 8–12 hpi were more than in the negative control group (Figures S1 and S2). The spherulocytes percentage, from 2–16 hpi, was more than in the control larvae and exhibited irregular fluctuation (Figures S3 and S4). The average of oenocytoids were less than one haemocyte (Figures S5 and S6).

### 3.4. General Protease Activity

**Figure 3:**
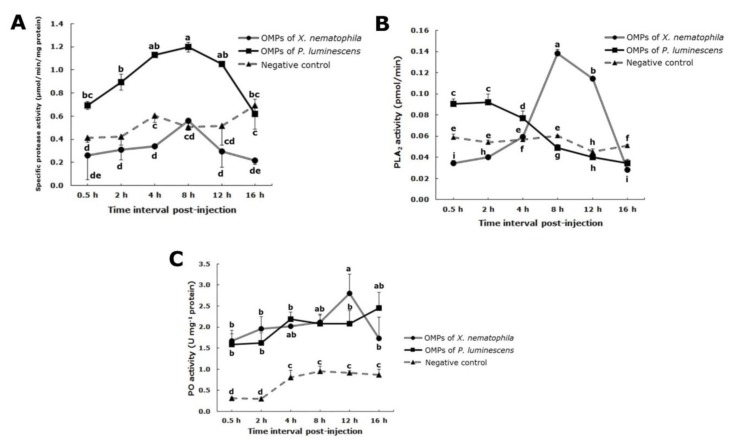
Changes in the patterns of humoral defense elements in fifth instar larvae of *Spodoptera exigua* after injection with outer membrane proteins of *Xenorhabdus nematophila* or outer membrane proteins of *Photorhabdus luminescens*, (**A**) protease, (**B**) phospholipase A2, (**C**) phenoloxidase. Each measurement consists of eight replications. The vertical bars represent the standard error of the means. Different letters above the error bars indicate a significant difference of interactive effect means between outer membrane proteins from the two bacterial species × interval times at α = 0.05 (Slicing test).

The total protease activity interactive effect between OMP types and intervals time was significant (F_10,54_ = 2.63, *p* < 0.05) for bacterial species both OMP (F_2,54_ = 68.06, *p* < 0.05) and time intervals (F_5,54_ = 4.72, *p* < 0.05) (Figure 3A). Larvae with OMPs of *P. luminescens* exhibited an increase protease activity from 0.5 hpi, reaching a maximizing level at 8 hpi, which then decreased. This activity was higher than in the negative control insects during this time interval (0.5 h: F_1,6_ = 28.12, *p* < 0.05; 2 h: F_1,6_ = 23.25, *p* < 0.05; 4 h: F_1,6_ = 77.99, *p* < 0.05; 8 h: F_1,6_ = 203.06, *p* < 0.05; 12 h: F_1,6_ = 9.71, *p* < 0.05; 16 h: F_1,6_ = 0.26, *p* > 0.05). *X. nematophila* OMPs, protease activity was statistically comparable with those in the control larvae, except for the absence of an increase at 4 hpi and a decline by 16 hpi (4 h: F_1,6_ = 24.44, *p* < 0.05; 16 h: F_1,6_ = 41.86, *p* < 0.05) (Figure 3A). Protease activity was significantly different between both OMPs types by 2–16 hpi (0.5 h: F_1,6_ = 4.02, *p* > 0.05; 2 h: F_1,6_ = 26.51, *p* < 0.05; 4 h: F_1,6_ = 2453.23, *p* < 0.05; 8 h: F_1,6_ = 46.58, *p* < 0.05; 12 h: F_1,6_ = 31.02, *p* < 0.05; 16 h: F_1,6_ = 8.61, *p* < 0.05). Larvae treated with both OMP types had the same pattern of increasing protease activity from 0.5 to a peak at 8 hpi and thereafter declining, albeit it at different rates.

### 3.5. Phospholipase A_2_ Assay

Interaction between OMPs and time intervals exerted a significant effect on PLA_2_ activity (Figure 3B). PLA_2_ activity in larvae with OMPs of *P. luminescens*, although constant from 0.5 to 2 hpi, thereafter decreased, and by 8 to 16 hpi the enzyme activity was less than the control. Larvae with *P. luminescens* OMPs exhibited statistically altered PLA_2_ activity compared with control larvae except at 12 hpi, at which time activities were similar (0.5 h: F_1,2_ = 32.23, *p* < 0.05; 2 h: F_1,2_ = 164.95, *p* < 0.05; 4 h: F_1,2_ = 287.99, *p* < 0.05; 8 h: F_1,2_ = 763.60, *p* < 0.05; 12 h: F_1,2_ = 862.95, *p* < 0.05; 16 h: F_1,2_ = 758.43, *p* < 0.05). In larvae with *X. nematophila* OMPs, the PLA_2_ level increased to a maximum value at 8 hpi and declined by 16 hpi to values less than the negative control levels (0.5 h: F_1,2_ = 494.29, *p* < 0.05; 2 h: F_1,2_ = 1977.05, *p* < 0.05; 4 h: F_1,2_ = 3580.53, *p* > 0.05; 8 h: F_1,2_ = 174.28, *p* < 0.05; 12 h: F_1,2_ = 369.49, *p* < 0.05; 16 h: F_1,2_ = 2763.84, *p* < 0.05) (Figure 3B). There was a significant difference between the effect of bacterial OMPs sources on PLA_2_ activity (0.5 h: F_1,2_ = 136, *p* < 0.05; 2 h: F_1,2_ = 54.13, *p* < 0.05; 4 h: F_1,2_ = 7.16, *p* > 0.05; 8 h: F_1,2_ = 325.54, *p* < 0.05; 12 h: F_1,2_ = 312.46, *p* < 0.05; 16 h: F_1,2_ = 2.12, *p* > 0.05); *P. luminescens* OMPs effect from 0.5 to 4 hpi being higher than for *X. nematophila* OMPs and the latter being greater than the former from 8–12 hpi. Control values were constant throughout the test times. Both source of OMPs inhibited PLA2 activity, the kinetic varying with the bacterial species. 

### 3.6. Phenoloxidase Assay

There was no evidence of significant interactive effect between OMP bacterial species and times on PO activity (F_10,54_ = 1.43, *p* > 0.05) (Figure 3C). Control larvae exhibited a marginal increase in PO activity by 4 hpi, followed by a plateau. The control PO values were significantly less than either OMP types at all sample times. There was no significant difference in PO activity in larvae with *P. luminescens* OMPs versus control from 0.5 to 16 hpi (0.5 h: F_1,6_ = 26.53, *p* < 0.05; 2 h: F_1,2_ = 28.33, *p* < 0.05; 4 h: F_1,2_ = 112.64, *p* < 0.05; 8 h: F_1,2_ = 55.42, *p* < 0.05; 12 h: F_1,2_ = 32.14, *p* < 0.05; 16 h: F_1,2_ = 31.45, *p* < 0.05). However, PO activity in larvae with *X. nematophila* OMPs increased gradually, reaching a maximum value by 12 hpi and then decreased (0.5 h: F_1,6_ = 29.66, *p* < 0.05; 2 h: F_1,2_ = 31.30, *p* < 0.05; 4 h: F_1,2_ = 983, *p* < 0.05; 8 h: F_1,2_ = 113.08, *p* < 0.05; 12 h: F_1,2_= 32.05, *p* < 0.05; 16 h: F_1,2_ = 7.20, *p* < 0.05) (Figure 3C). There was no significant difference between OMPs of *X. nematophila* and *p. luminescens* on PO activation (0.5 h: F_1,6_ = 0.05, *p* > 0.05; 2 h: F_1,6_ = 0.77, *p* > 0.05; 4 h: F_1,6_ = 1.11, *p* > 0.05; 8 h: F_1,6_ = 0.03, *p* > 0.05; 12 h: F_1,6_ = 1.75, *p* > 0.05; 16 h: F_1,6_ = 1.34, *p* > 0.05). 

### 3.7. Attacin Gene Expression

**Figure 4:**
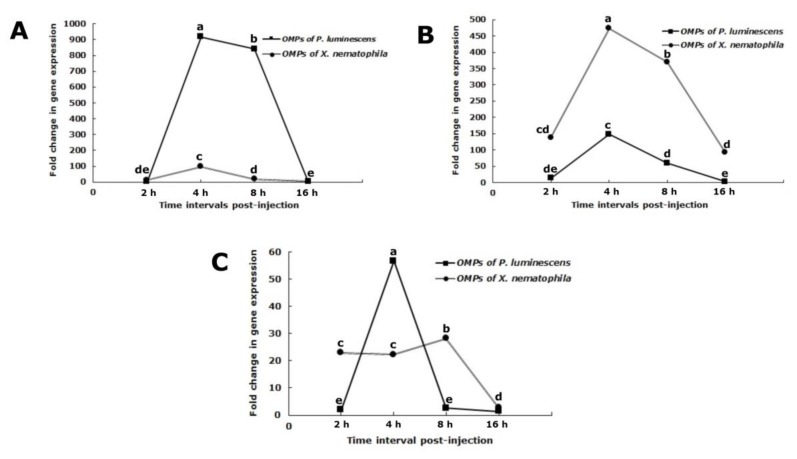
Changes in the patterns of the expression of selected inducible antimicrobial genes in fifth instar larvae of *Spodoptera exigua* after injection with outer membrane proteins of *Xenorhabdus nematophila* or outer membrane proteins of *Photorhabdus luminescens*, (**A**) attacin, (**B**) cecropin, (**C**) spodoptericin. Each measurement consists of eight replications. The vertical bars represent the standard deviations of the means. Different letters above the error bars indicate a significant difference of interactive effect means between outer membrane proteins types × interval times at α = 0.05 (Slicing test).

The effect of OMPs of *P. luminescens* and *X. nematophila* on fluctuation of the attacin expression in *S. exigua* larvae was significant (F_1,8_ = 25,737.1, *p* < 0.05) and varied with the bacterial species (Figure 4A). In larvae with *P. luminescens* OMPs, attacin expression value gradually increased from 2 hpi to a maximum level at 4 hpi (4 h: F_1,2_ = 9446, *p* < 0.05) then decreased by 8 hpi (16 h: F_1,2_ = 3.36, *p* > 0.05). Attacin expression in larvae with *X. nematophila* OMPs was less than those with OMPs of *P. luminescens* and reached a maximum level with 96.86 ± 1.14-fold greater than the negative control by 2 hpi. Thus, the attacin gene was upregulated by OMP of both bacterial species, but the degree of upregulation varied with the source of total OMP. The gene expression in injected larvae with control buffer was 0.39–0.5 times higher than the normal sample (non-injected larvae). There was a significant difference between the effect of both bacterial species on attacin expression (2 h: F_1,2_ = 477.80, *p* < 0.05; 4 h: F_1,2_ =9308, *p* < 0.05; 8 h: F_1,2_ = 6009.70, *p* < 0.05; 16 h: F_1,2_ = 1403.56, *p* < 0.05) (Figure 4A). The total OMPs from both bacterial species were able to decrease attacin expression.

### 3.8. Cecropin Gene Expression

There were significant fluctuations of cecropin expression in *S. exigua* larvae with different OMPs sources over time (F_1,8_ = 1706, *p* < 0.05) (Figure 4B). Also, interactive effects between both bacterial OMPs and time (F_3,8_ = 141.32, *p* < 0.05) in larvae with *P. luminescens* OMPs, cecropin expression elevated gradually from 2 hpi to the highest level at 4 hpi (4 h: F_1,2_ = 1921.02, *p* < 0.05) and then decreased (8 h: F_1,2_ = 0, *p* > 0.05; 16 h: F_1,2_ = 38.27, *p* < 0.05). The expression pattern of the cecropin gene in larvae with *X. nematophila* OMPs was similar to those of *P. luminescens* OMPs. However, cecropin expression in larvae with OMPs *X. nematophila* was less than those of treated with *P. luminescens* OMPs. The cecropin expression in injected larvae with both OMPs was positive. However, the ability of OMPs of *X. nematophila* to decrease cecropin expression was more than *P. luminescens* OMPs from 4 to 16 hpi. The gene expression in larvae injected with control buffer was 0.03–0.05 times higher than the normal sample. There was a significant difference between OMPs of *X. nematophila* and *P. luminescens* on cecropin expression at 2 and 16 hpi (2 h: F_1,2_ =65.8, *p* < 0.05; 4 h: F_1,2_ = 169.12, *p* < 0.05; 8 h: F_1,2_ = 143.08, *p* < 0.05; 16 h: F_1,2_ = 59.02, *p* < 0.05) (Figure 4B). Both OMPs types were able to decrease cecropin expression.

### 3.9. Spodoptericin Gene Expression

There was a significant interactive effect between both bacterial OMPs and time (F_3,8_ = 289.26, *p* < 0.05). Also, bacterial OMPs (F_1,3_ = 17.90, *p* < 0.05) during different times (F_3,8_ = 387.15, *p* < 0.05) significantly affected the expression of spodoptericin (Figure 4C). Spodoptericin expression in larvae with *P. luminescens* OMPs compared with the negative control reached the maximum level by 4 hpi (4 h: F_1,2_ = 873.98, *p* < 0.05) and then decreased (8 h: F_1,2_ = 10.45, *p* > 0.05; 16 h: F_1,2_ = 47.82, *p* < 0.05). In larvae with *X. nematophila* OMPs, a gradual decrease of spodoptericin occurred from 8 to 16 hpi (16 h: F_1,2_ = 3.15, *p* > 0.05). The change of spodoptericin gene activity was positive during early expose to the protein, revealing that gene is up-regulated (Figure 4C). Then, both bacterial OMPs were able to decrease spodoptericin expression. The trend of induced spodoptericin in larvae with *X. nematophila* OMPs and *P. luminescens* OMPs differed. 

## 4. Discussion

In the current study, the cellular and humoral aspects of the immune system in *S. exigua* larvae against purified total OMPs of *X. nematophila* and *P. luminescens* were uniquely considered. The results imply that OMPs of the bacteria were able to modulate both the cellular and humoral defenses, the different OMP responses representing different types or amounts of the modulating components from the two bacteria species. The OMPs of these insect pathogenic bacteria likely contribute to their virulence. The OMPs of mammalian pathogenic bacteria serve as virulence elements for the evasion of the immune of the host [7]. Interesting and puzzling is that gene expression in the present recent study was influenced sooner than cellular and early stage humoral factors, even though humoral cytokines activate traditional cellular responses by influencing cellular signaling pathways, and elicit AMPs production independently or after host cellular responses [25]. THC and granulocyte levels of *S. exigua* after injection of *X*. *nematophila* OMPs and *P. luminescens* OMPs decreased at different times early in the post inoculation phase. This could be attributed to LPS-contaminated OMPs eliciting apoptotic symptoms in the haemocytes by of the releasing of LPS [26]. The decline in the density of all haemocyte types in *Galleria mellonella* Linneaus larvae resulted from the lipid A moiety of *X*. *nematophila* and *P. luminescence* LPS action triggering haemocytes lysis (including oenocytoids) and inhibiting PO activation but not activity [27]. Herein, OMP components activate PO directly or indirectly by lysis oenocytoids, and releasing the enzyme, as reported for *S. exigua* [28]. However, unlike the effect of LPS on *G. mellonella*, in S. exigua with OMPs, there was no correlation between PO activity and oenocytoid density. In larvae with OMPs of both bacterial species, PO activity increased over the incubation time, but in the treatments with *X. nematophila* OMPs it decreased after the peak. We assume the decrease PO in larvae with *X. nematophila* OMPs was caused by OMPs. Collectively, the effects of OMPs on the hemocytes and continuous activation of PO imply that there were no physiological amounts of LPS on the purified OMPs in the present study. Comparing protease activity in OMP treated larvae with the negative larva controls was activated over time using OMP from both bacterial types, the magnitude being greater for *P*. *luminescence* than *X. nematophila*, the latter being similar to the control. In treated larvae with OMPs of *X*. *nematophila*, the protease activity at the beginning of injection was less than the negative control. 

Park and Kim [11] and Park et al. [10] confirmed that intact *X. nematophila* in *S. exigua* was able to decrease PLA_2_ activity, limiting cellular immunity. In the present study, PLA_2_ activity relative to the constant control levels increased in the early post-inoculation stages and decreased after maximum activity later in the cycle. The differences could be related to variations in the types of OMP components, their amounts, biological properties, and spatial organization in the total OMP extracts. Here it is confirmed that protein profiles of *X. nematophila* and *P. luminesces* are different. The lower ability of *P. luminescens* OMPs on haemocytes destruction, inhibition of PO activity, and decrease of some humoral elements in initial times are in accordance with Forst and Nealson [8], who indicated as in the present work, that the surface of *Photorhabdus* spp. may be different from of *Xenorhabdus* spp. The importance of cell surface properties in the life cycle and phase variation of *Xenorhabdus* cells has been linked to identified outer membrane proteins such as fimbria (pilin) and flagella [29]. Although the properties of OMPs, flagella in phase I and phase II cells, fimbria (pilin) protein of surveyed *X. nematophila* are known [30], the cell surface properties of *Photorhabdus* cells have been more limited to OMPs [8,31,32].

Here it is reported that in *S. exigua* larvae the AMPs attacin, cecropin, and spodoptericin genes are activated by the OMPs. Bacterial LPS activates numerous types of AMPs in Lepidoptera [33]. While OMPs and LPS effects may confer resistance to the bacteria by the host insect, Duperthuy et al. [34] established that outer membrane protein U (OmpU) of *Vibrio splendidus*, the oyster pathogenic bacterium, contributes to its virulence by making the bacterium resistant to antimicrobial peptides. Vanaja et al. [35], reported that OMVs of extracellular Gram-negative mammalian bacteria can deliver LPS into the host cells; however, the mechanism of LPS translocation remains unclear.

Herein, OMPS of both *X. nematophila* and *P. luminescens* upregulated attacin and cecropin for the expression of these AMPs by *X. nematophila* OMPs being less than *P*. *luminescens*. This phenomenon may be due to differences in OMP composition. Spodoptericin is expressed in lepidopteran insects with Gram-positive bacteria [36]. In the present work, spodoptericin was expressed after injection of OMPs from both Gram-negative bacteria.

The pronounced irregulars’ fluctuations of attacin and cecropin occurred in larvae with OMPs of *X. nematophila*. Whereas, AMPs expression in larvae with *P. luminescens* OMPs reached the maximum level by 4 hpi and then decreased. According to Castillo et al. [37], the expression level of cecropinA1/A2 in *Drosophila* after infection by *Photorhabdus* decreased at 30 h. The decrease in AMPs expression could reflect the ability of *Photorhabdus* to degrade the host AMPs. Of the cytotoxic phospholipase C, protease, proelastase, and hemolysins in OMVs of *P. aeruginosa, Proteus mirabilis*, and *Serratia marcescens* [6], known to destroy the membrane of eukaryotic cells [38]. Insect AMPs are synthesized after direct and indirect recognition of pathogens by the haemocytes [39] and fat bodies [40]. However, destruction of haemocytes by OMPs may have partly led to a reduction in AMPs expression. *P. luminescens* OMPs decreased haemocyte density, after which AMPs expression was limited. *X. nematophila* OMPs elicited total heamocyte population that was initially less than *P. luminescens*, as was the overall AMPs expression. 

Herein, attacin expression was more than cecropin and spodoptericin, the latter, two AMPs possibly being digested by bacterial protease. Purified AMP peptides from *G. mellonella* (Gm anionic peptide, Gm proline–rich peptide, defensin, a defensin-like and cecropin D-like) and cecropin B from *Hyalophora cecropia* Linnaeus were sensitive to degradation by elastase B from *P. aeruginosa* [41,42,43]. The secreted alkaline metalloprotease (PrtA) produced by *Photorhabdus* sp. has homologies in *P. luminescens* and *P. temperata* that collectively inhibit the activity of *G*. *mellonella* cecropins A and B [44]. *Photorhabdus* PrtS also cleaves insect antibacterial peptides [45]. The alkaline protease of *P. aeruginosa* may also be responsible for some degradation/inactivation of inducible AMPs in *G. mellonella* [42]. Live *Xenorhabdus* inhibits expression of lysozyme and [46], cecropin in *S. exigua* [47] and purified protease II from *X. nematophila* reduced 97% of cecropin A [48]. 

Generally, humoral and cellular immune defenses of insects are cross-linked. The PLA_2_ has an important role in eicosanoid biosynthesis of insects, the eicosanoids affecting aggregation of haemocytes, haemocyte migration, and release of prophenoloxidase from oenocytoids [49]. Thus, the effect of PLA_2_ activity leads to a change in cellular and humoral reactions. Here, the OMPs of *X. nematophila* and *P. luminescens* decreased PLA_2_ activity and probably prevented eicosanoid biosynthesis, since AMPs expression in *S. exigua* by eicosanoid pathway is inhibited by intact *X. nematophila* [50]. 

Herein, the data about the involvement of OMPs of *X. nematophila* and *P. luminescens* on *S. exigua* haemocytes and the expression pattern of main AMPs during are novel for insect pathology. We proposed the role for OMPs in the destruction of haemocytes, modulation of plasma enzymes (PLA_2_ and PO) as the main defense source of infected insect. Here, in addition to the haemocyte density and PO activity at interval times, the differential effects of OMPs from *X. nematophila* and *P. luminescens* on the number of granulocytes, protease, PLA_2_ activities, attacin, cecropin, and spodoptericin expression were surveyed and indicated the difference in likely virulent factors between the bacterial species. 

In summary, cumulative information suggests that secretion of insect toxins, outer membrane proteins, other extracellular products, and the release of LPS molecules from the bacterial envelope lead to the death of the host. Also, the current work increased our knowledge about the ability of OMPs in the suppression of cellular and humoral defense of insects.

## 5. Conclusions

This study provides a novel insight into some aspects of immune defense of *S. exigua* larvae against outer membrane proteins of *X. nematophila* and *P. luminescens*. The obtained results indicated that OMPs of symbiotic bacteria affected on cellular and humoral immune system. But there were differences between them. The current work increased us knowledge about the pathogenicity of *X*. *nematophila* and *P. luminescens*.

## Supplementary Materials

The following are available online at https://www.mdpi.com/2075-4450/10/10/352/s1, Figure S1. Changes of oenocytoids in fifth instar larvae of *Spodoptera exigua* after injection with outer membrane proteins of *Photorhabdus luminescens.* Figure S2. Chnages of oenoctyoids in last instar larvae of *Spodoptera exigua* after injection with outer membrane proteins of *Xenorhabdus nematophil.* Figuree S3. Fluctuation pattern of plasmatocytes in fifth instar larvae of *Spodoptera exigua* after injection with outer membrane proteins of *Photorhabdus luminescens.* Figure S4 Changes of plasmatocytes in the last larvae of *Spodoptera* exigua after injection with outer membrane proteins of *Xenorhabdus nematophil.* Figure S5. Changes of spherulocytes in fifth instar larvae of *Spodoptera exigua* after injection with outer membrane proteins of *Photorhabdus luminescens.* Figure S6. Fluctuation pattern of spherulocytes in last instar larvae of *Spodoptera exigua* after injection with outer membrane proteins of *Photorhabdus luminescens.*
