# Supplementary material for: Functional Characterization of Outer Membrane Proteins (OMPs) in Xenorhabdus nematophila and Photorhabdus luminescens through Insect Immune Defense Reactions
Source: Insects. 2019 Oct 17;10(10):352. doi: 10.3390/insects10100352 (PMC6835289; doi:10.3390/insects10100352)
Supplement: Supplementary file 1 [file insects-10-00352-s001.pdf]

insects

Article

Functional Characterization of Outer Membrane Proteins (OMPs) in *Xenorhabdus nematophila* and *Photorhabdus luminescens* through Insect Immune Defense Reactions

Reyhaneh Darsouei [1], Javad Karimi [1,*], and Gary B. Dunphy [2]

[1] Biocontrol and Insect Pathology Lab., Department of Plant Protection, School of Agriculture, Ferdowsi University of Mashhad, Mashhad 9177948974, Iran; rdarsouei@gmail.com
[2] Department of Natural Resource Sciences, Faculty of Agricultural and Environmental Sciences, McGill University, 21 111 Lakeshore Rd, Quebec H9X 3V9, Canada; gary.dunphy@mcgill.ca
* Correspondence: jkb@um.ac.ir; Tel.: +98-51-3880-5817; Fax: +98-51-37430-878

Received: 10 September 2019; Accepted: 6 October 2019; Published: date

Supplementary Materials

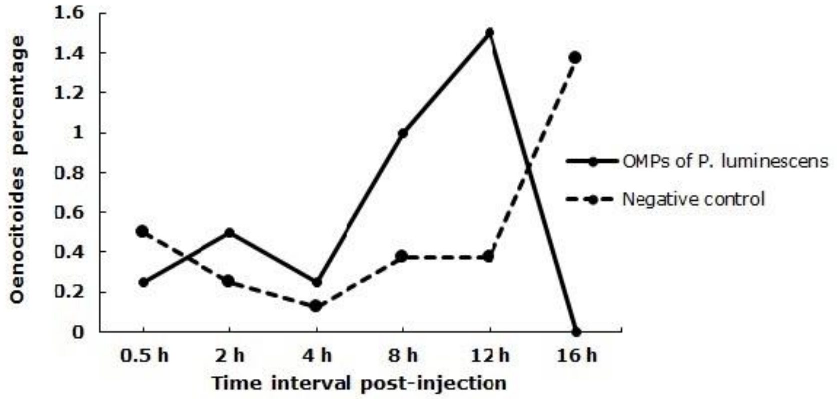

Figure S1. Changes of oenocytoids in fifth instar larvae of *Spodoptera exigua* after injection with outer membrane proteins of *Photorhabdus luminescens.*

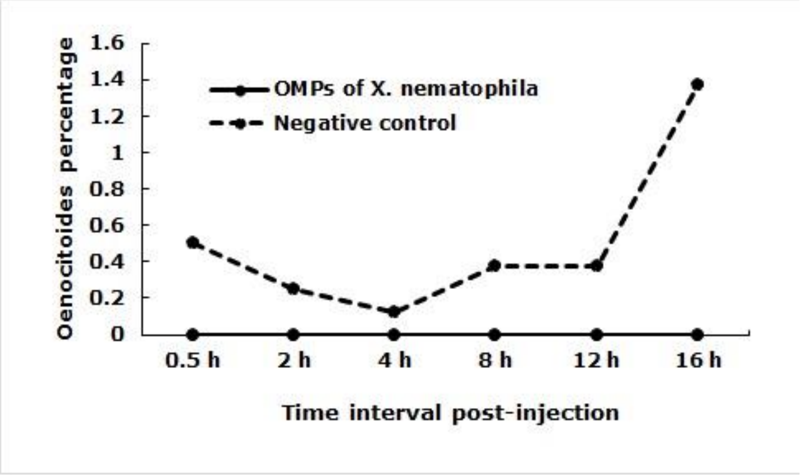

Figure S2. Chnages of oenoctyoids in last instar larvae of *Spodoptera exigua* after injection with outer membrane proteins of *Xenorhabdus nematophil.*

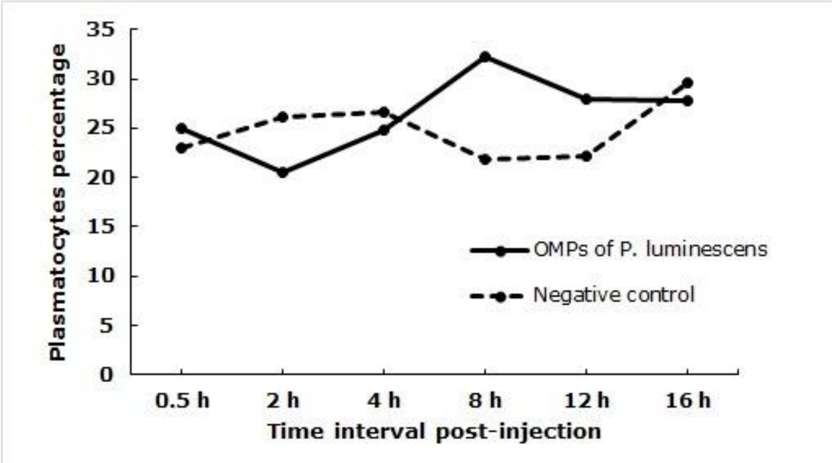

Figure S3. Fluctuation pattern of plasmatocytes in fifth instar larvae of *Spodoptera exigua* after injection with outer membrane proteins of *Photorhabdus luminescens.*

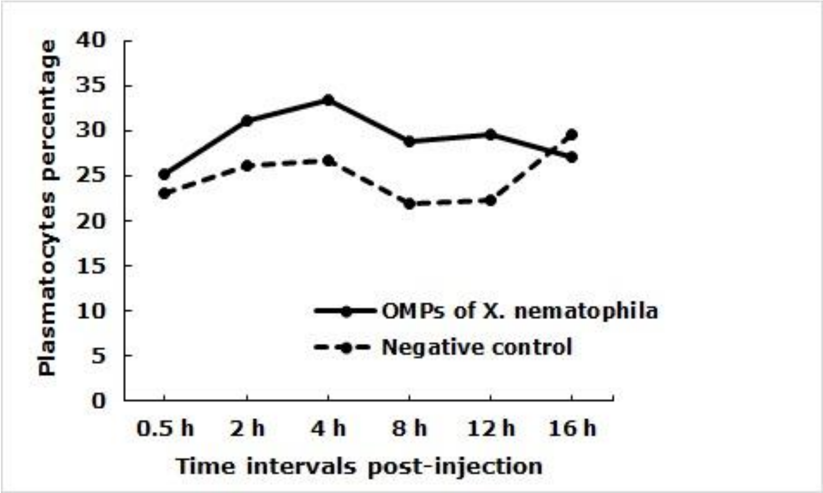

Figure S4. Changes of plasmatocytes in the last larvae of *Spodoptera* exigua after injection with outer membrane proteins of *Xenorhabdus nematophil.*

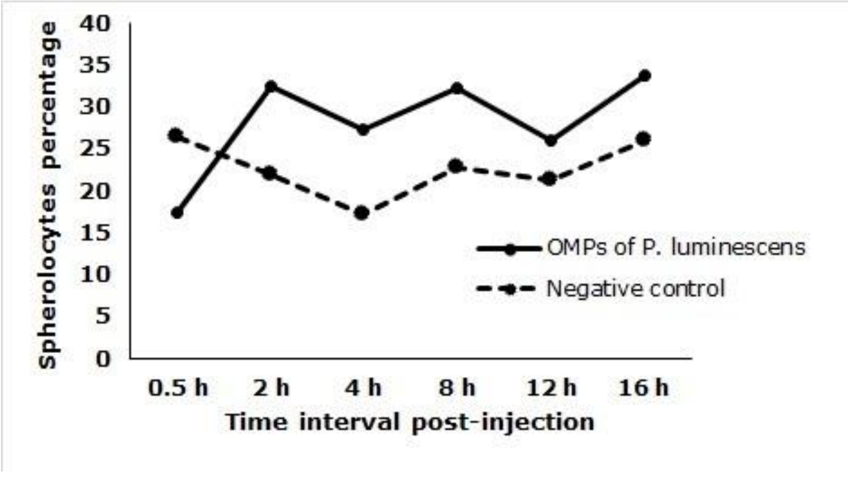

Figure S5. Changes of spherulocytes in fifth instar larvae of *Spodoptera exigua* after injection with outer membrane proteins of *Photorhabdus luminescens.*

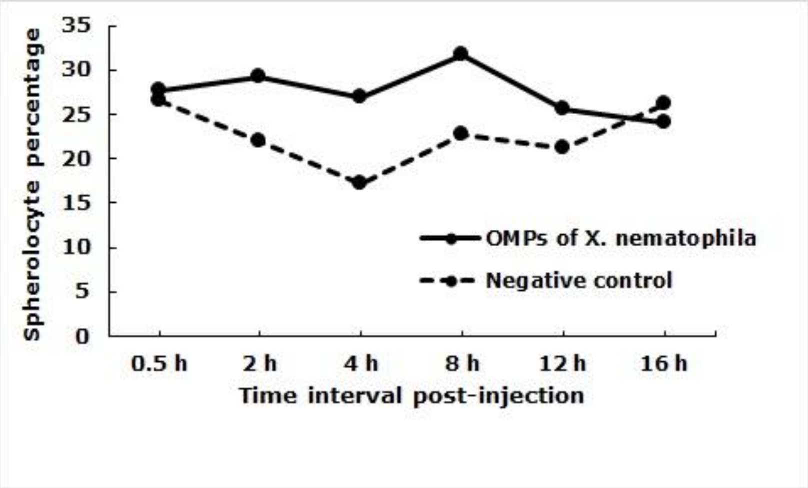

Figure S6. Fluctuation pattern of spherulocytes in last instar larvae of *Spodoptera exigua* after injection with outer membrane proteins of *Photorhabdus luminescens*.
